# On the Conductivity of Proton-Exchange Membranes Based on Multiblock Copolymers of Sulfonated Polysulfone and Polyphenylsulfone: An Experimental and Modeling Study

**DOI:** 10.3390/polym13030363

**Published:** 2021-01-23

**Authors:** Nieves Ureña, M. Teresa Pérez-Prior, Belén Levenfeld, Pablo A. García-Salaberri

**Affiliations:** 1 Departamento de Ciencia e Ingeniería de Materiales e Ingeniería Química, IAAB, Universidad Carlos III de Madrid, 28911 Leganés, Spain; murena@ing.uc3m.es (N.U.); maperezp@ing.uc3m.es (M.T.P.-P.); bll@ing.uc3m.es (B.L.); 2 Departamento de Ingeniería Térmica y de Fluidos, Universidad Carlos III de Madrid, 28911 Leganés, Spain

**Keywords:** ionic conductivity, water uptake, multiblock copolymer, percolation theory, modeling, characterization, proton-exchange membrane

## Abstract

The effect of relative humidity (RH) and degree of sulfonation (DS) on the ionic conductivity and water uptake of proton-exchange membranes based on sulfonated multiblock copolymers composed of polysulfone (PSU) and polyphenylsulfone (PPSU) is examined experimentally and numerically. Three membranes with a different DS and ion-exchange capacity are analyzed. The heterogeneous structure of the membranes shows a random distribution of sulfonated (hydrophilic) and non-sulfonated (hydrophobic) domains, whose proton conductivity is modeled based on percolation theory. The mesoscopic model solves simplified Nernst–Planck and charge conservation equations on a random cubic network. Good agreement is found between the measured ionic conductivity and water uptake and the model predictions. The ionic conductivity increases with RH due to both the growth of the hydrated volume available for conduction and the decrease of the tortuosity of ionic transport pathways. Moreover, the results show that the ionic conductivity increases nonlinearly with DS, experiencing a strong rise when the DS is varied from 0.45 to 0.70, even though the water uptake of the membranes remains nearly the same. In contrast, the increase of the ionic conductivity between DS=0.70 and DS=0.79  is significantly lower, but the water uptake increases sharply. This is explained by the lack of microphase separation of both copolymer blocks when the DS is exceedingly high. Encouragingly, the copolymer membranes demonstrate a similar performance to Nafion under well hydrated conditions, which can be further optimized by a combination of numerical modeling and experimental characterization to develop new-generation membranes with better properties.

## 1. Introduction

Fuel cells (FCs) are electrochemical devices that convert fuels into electric power in an efficient and environmentally friendly way. They are compatible with renewable sources and modern energy carriers, i.e., hydrogen, for sustainable development in a wide range of applications, including transportation, stationary electricity generation, and portable power devices [1,2,3]. Hence, FCs are expected to play a key role as power sources in this century, providing clean, efficient, and flexible chemical-to-electrical energy conversion.

Among the various types of FCs, proton-exchange membrane fuel cells (PEMFCs) have drawn significant attention as alternative clean energy conversion devices because of their high efficiency, low operating temperature, fast start-up, and their potential to operate with fuels from renewable sources [4,5]. In these devices, the proton-exchange membrane (PEM) is an efficiency-determining component, which allows the transport of protons and separates the cell compartments [6]. However, before large-scale commercialization of PEMFCs, some issues associated with the polymer electrolyte must be addressed. One of the main drawbacks of PEMFC technology is the need to operate at high relative humidity (RH) in order to achieve good proton conductivity, since mass-transport losses at high RH decrease performance and the use of external humidification complicates the PEMFC system [7,8,9,10]. In addition, excessive swelling of functionalized polymers with increased water sorption can lead to cell failure [11]. Therefore, there is a need to design PEMs that can offer high proton conductivity and stable operation at reduced RH, i.e., RH% < 50 [12].

Generally, PEMs are composed of a polymer backbone with acid-bearing functionalities (e.g., sulfonic acid groups). The current state-of-the-art PEMs are based on perfluorinated sulfonic-acid (PFSA) membranes, such as Nafion [13]. Nafion is widely known for its remarkable proton conductivity and excellent chemical and mechanical properties at fully hydrated state [14]. However, Nafion has high manufacturing cost, low proton conductivity at temperatures above 80 °C, and high crossover rates [15,16,17]. The development of PEMs with microphase-separated morphology, i.e., including well-separated hydrophilic ion-conducting and hydrophobic phases, has been considered as the most relevant strategy to increase ionic conductivity by enhancing ion mobility and ion concentration [18,19]. On this matter, self-assembled block copolymers can lead to well-defined structures where the morphology and domain size are tunable [20]. One of the blocks is sulfonated to facilitate ionic conduction, while the other block remains unaltered to provide mechanical stability. Relevant works published on copolymer-based PEMs were presented by Yoo et al. [21], Jung et al. [22], and Bae et al. [23]. Yoo et al. [21] synthesized poly(arylene ether) (PAE) multiblock copolymers with densely sulfonated hydrophilic blocks and well-separated phase morphology. As a result, high proton conductivity (15 mS cm^−1^) at 80 °C and moderate RH (50%), comparable to Nafion 212 (25 mS cm^−1^) was achieved. A similar copolymer structure was studied by Jung et al. [22]. They prepared multiblock copolymers combining hydrophilic sulfonated poly(arylene sulfone) (SPAS) blocks and hydrophobic poly(arylene ether sulfone) (PAS) blocks. The membranes showed well-connected hydrophilic nanophase domains, even though the water sorption capacity of the membranes was out of control in some cases. The proton conductivity at 80 °C and 50% RH was 28 mS cm^−1^. In this line, Bae et al. [23] synthesized poly(arylene ether sulfone ketone) (SPESK) multiblock copolymer membranes with highly interconnected sulfonated hydrophilic blocks, which showed a good proton conductivity of 30 mS cm^−1^ at 80 °C and 40% RH (ion-exchange capacity, $IEC=1.87{\;\mathrm{meq}\;g}^{-1}$). In the case of copolymer membranes of sulfonated polysulfone (SPSU) and polyphenylsulfone (SPPSU), the proton conductivity at 80 °C and 50–60% RH is around 10 mS cm^−1^ when both segments are sulfonated in a similar degree ($IEC=1.64{\;\mathrm{meq}\;g}^{-1}$) [24]. Further improvements can be achieved by a combination of experimental and numerical work [25,26,27].

A large body of modeling work can be found in the literature dealing with ion conduction in PEMs, with a special focus on Nafion and similar PFSA membranes. Mathematical modeling of ion transport in PEMs poses a complex multiscale problem, which occurs within a dynamic framework with varying morphology and transport properties [28,29,30,31,32,33,34,35,36]. Eikerling et al. [37] presented a random network model of charge transport in PEMs based on effective medium theory to examine the membrane complex impedance as a function of water content. They found that the conductivity showed a quasi-percolation type dependence with water content, growing above a certain critical level, whereas the geometrical capacity either increased with water content or reached a maximum at the percolation threshold, depending on the model parameters. In a subsequent work, Eikerling et al. [38] developed a phenomenological model of proton conduction in PEMs accounting for proton transfer in condensed media and heterogeneous membrane structure. The combination of proton transfer processes in a single pore with the global pore-network behavior allowed them to relate structural and kinetic characteristics of PEMs, obtaining good agreement with typical experimental data. Weber and Newman [39,40] presented a comprehensive physical and mathematical model of transport in PEMs, built upon the wealth of knowledge contained in the literature. The predictions of their capillary cluster-network model were in good agreement with previous experimental data. Among other conclusions, the model provided an explanation of Schroeder’s paradox, thus bridging the gap between one-phase (vapor-equilibrated membranes) and two-phase (liquid-equilibrated membranes) models previously found in the literature. Tongwen et al. [41] presented a three-phase model based on percolation theory, i.e., accounting for the pure-gel phase (active region), inert-gel phase (inactive region), and the inter-gel phase (the interstitial region between the other two regions). Their results highlighted the percolative nature and the importance of cluster connectivity on ionic conduction in sulfonated poly(phenylene oxide) (SPPO) membranes. Gostick and Weber [42] developed a resistor and pore-network model to examine proton transport in various ion-conducting polymers (block copolymers, Nafion and thin ionomer films). The model developed was shown to be an efficient tool to study transport in PEMs from the nanoscale morphology through the mesoscale transport pathways to the observable macroscale properties. More recently, Zhang et al. [43] presented a novel model, which combined a simplified three-phase representation of the membrane with percolation theory, to study both conductivity and permselectivity of SPPO membranes. Their model successfully explained the decrease in permselectivity with decreasing membrane thickness considering the membrane void ratio and percolative state of the lattice structure.

In this work, alternative PEMs from Nafion based on sulfonated multiblock copolymers of polysulfone (PSU) and polyphenylsulfone (PPSU) segments have been characterized and modeled simultaneously for the first time. Field emission scanning electron microscopy (FE-SEM) was used to evaluate the morphology of the membranes. Considering the original design of the material endowed with high entanglement level and the mechanical and electrochemical properties, the behavior of the material cannot be only known through experimental data. Thus, measured proton conductivity and water absorption capacity of the copolymer membranes have been modeled with a mesoscopic model based on percolation theory to provide fundamental information on the behavior of the membranes. The organization of the paper is as follows. In Section 2, the synthesis and experimental characterization of the membranes are presented. In Section 3, the mathematical formulation and implementation of the numerical model are described. In Section 4, the results are discussed, including a comparison between the experimental and numerical data in terms of proton conductivity and water uptake. Finally, the conclusions and future work are presented in Section 5.

## 2. Experimental

### 2.1. Materials

**Figure 1:**
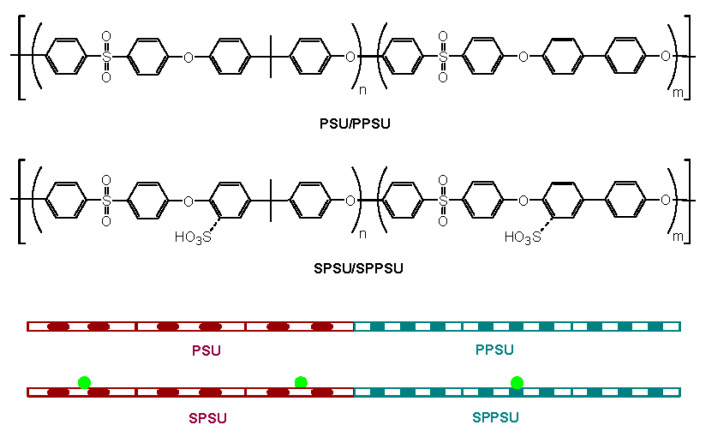
Chemical structure and schematic representation of polysulfone/polyphenylsulfone (PSU/PPSU) and sulfonated polysulfone / polyphenylsulfone (SPSU/SPPSU) copolymers. Sulfonic groups are represented by green circles.

PSU/PPSU poly(ether sulfone)s (PES) multiblock copolymer was synthesized via polycondensation using a “one-pot two-step synthesis” of commercial monomers, as described in previous work [24]. The chemical structure of the non-sulfonated and sulfonated copolymers is shown in Figure 1. Sulfonation reaction of copolymers was performed by reaction with trimethylsilyl chlorosulfonate (TMSCS) according to the procedure described by Chao et al. [44]. It should be pointed out that chlorosulfonic acid, sulfur trioxide/triethyl phosphate complex, and TMSCS are usually used as sulfonating agents. However, TMSCS was chosen here to carry out the sulfonation of the multiblock copolymers since this sulfonating agent causes a lower degradation of the polymer chain [45]. Subsequently, polymer membranes with thicknesses in the range of 50–75 μm were prepared. Membranes in the acidic form were obtained by immersion of the membranes in the Na^+^ form in a 1 M HCl solution at 60 °C for 24 h. The density of the membranes was measured using samples, whose dimensions and weights were determined after drying at 60 °C under vacuum [46]. The average density of the dry membranes was found to be $\rho_{\mathrm{dry}}\approx 1140{\;\mathrm{kg}\;m}^{-3}$, which is in the range of PSU (1240 ${\mathrm{kg}\;m}^{-3}$), PES (1370 ${\mathrm{kg}\;m}^{-3}$), and PPSU (1290 ${\mathrm{kg}\;m}^{-3}$) [47]. This value is lower than that commonly found in PFSA membranes, such as Nafion (1170–1980 ${\mathrm{kg}\;m}^{-3}$) [30]. Hereafter, membranes are abbreviated as SPES followed by a number depending on the PSU unit:TMSCS molar ratio used in the synthesis. Thus, membranes SPES 1, SPES 2, and SPES 3 correspond to 1:3, 1:6, and 1:9 molar ratios, respectively, having different average degrees of sulfonation (DS).

### 2.2. Characterization: Degree of Sulfonation, Ion-Exchange Capacity, Morphology, Water Uptake, and Ionic Conductivity

Nuclear magnetic resonance (^1^H-NMR) spectra were registered at 300.12 MHz on a Bruker WM 250 spectrometer [24]. ^1^H-NMR spectrum of sulfonated copolymers showed that the peak associated with the protons adjacent to the attached sulfonic groups was upshifted in both segments. However, the peak of the PSU unit was overlapped by the peaks associated with the PPSU block, impeding the determination of the DS of the PSU unit. The  DS of the PPSU unit was calculated through ^1^H-NMR, whereas the DS of the PPSU unit was determined from the IEC (see Table 1). Considering the definition of IEC:

(1) $$\begin{array}{c} IEC=\frac{N_{{\mathrm{SO}}_{3}^{-}}}{m_{p}};\;N_{{\mathrm{SO}}_{3}^{-}}=nDS_{\mathrm{PSU}}+mDS_{\mathrm{PPSU}};\;m_{p}=M_{b}+nDS_{\mathrm{PSU}}M_{{\mathrm{SO}}_{3}^{-}}+mDS_{\mathrm{PPSU}}M_{{\mathrm{SO}}_{3}^{-}} \end{array}$$

The DS of the PSU unit is given by

(2) $$DS_{\mathrm{PSU}}=\frac{IEC\left(M_{b}+mDS_{\mathrm{NMR},\mathrm{PPSU}}M_{{\mathrm{SO}}_{3}^{-}}\right)-mDS_{\mathrm{NMR},\mathrm{PPSU}}}{n\left(1-IECM_{{\mathrm{SO}}_{3}^{-}}\right)\;}$$

where $N_{SO_{3}^{-}}$ and $m_{p}$ are the number of moles of sulfonic groups ${\mathrm{SO}}_{3}^{-}$ and mass of sulfonated copolymer per mol of dry sulfonated copolymer, n and m are the number of structural units (molecules) of the PSU and PPSU blocks, and $M_{b}$ and $M_{SO_{3}^{-}}$ are the molecular masses of the polymer backbone and sulfonic group, respectively.

Using the DS of the two blocks, the average DS of the membranes, i.e., the percentage of sulfonated blocks of the copolymer membrane, was determined as

(3) $$DS=\frac{nDS_{\mathrm{PSU}}+mDS_{\mathrm{PPSU}}}{n+m}$$

According to this definition, DS is bounded between 0 and 1. The limit DS=0 corresponds to a non-sulfonated membrane, while the limit DS=1 corresponds to a fully sulfonated membrane (i.e., $DS_{\mathrm{PSU}}=DS_{\mathrm{PPSU}}=1$). It should be noted that DS is approximately equal to the volume fraction of sulfonated sites of the membrane, since the volumes of the non-sulfonated and sulfonated blocks are similar because of their similar chemical structure, i.e., $V_{\mathrm{PSU}}\approx V_{\mathrm{PPSU}}\approx V_{\mathrm{SPSU}}\approx V_{\mathrm{SPPSU}}$ [41].

The IEC was determined by both acid–base titration in aqueous solution and titration in an organic solvent (see [24] for further details).

**Figure 2:**
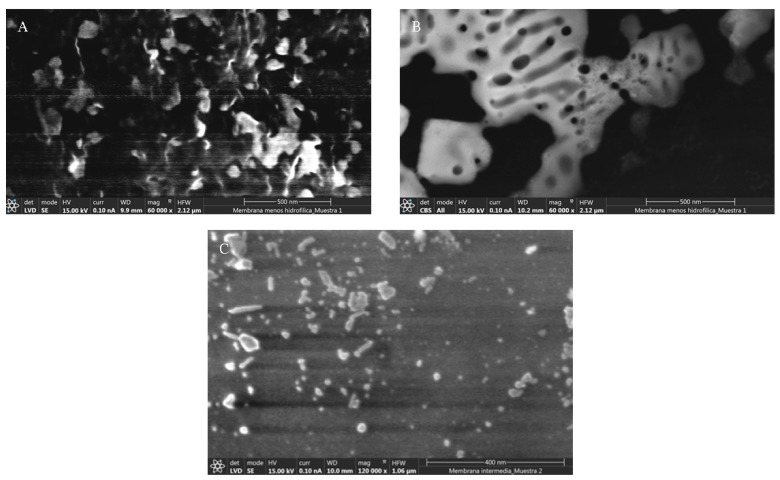
FE-SEM images of the cross section of SPES 1 (**A**), and the surfaces of SPES 1 (**B**) and SPES 2 (**C**) membranes.

The morphology of the membranes was characterized by FE-SEM using a FEI TENEO-LoVac equipped with an energy-dispersive detector (EDS-EDAX). The images were recorded at 5–10 kV. Mobile protons attached to sulfonic groups were replaced by Pb^2+^ ions for a better visualization of the hydrophilic domains. To this end, dried membranes in Na^+^ form were immersed in a 1 M HCl solution several times for 48 h to replace Na^+^ with H^+^ and then washed with deionized water. The resulting membranes were immersed in a 1 M Pb(NO_3_)_2_ solution stirred for 48 h, and dried under vacuum at 60 °C. Figure 2 shows representative FE-SEM images of the morphology of the membranes, corresponding to cross-sections of SPES 1 (A) and the surfaces of SPES 1 (B) and SPES 2 (C). As can be seen, the copolymer membranes present a heterogeneous distribution of hydrophilic and hydrophobic domains. The lighter zones correspond to regions with higher hydrophilicity due to a larger content of sulfonic groups (i.e., presence of Pb^+2^ as a counter-ion of sulfonic groups). These regions are prone to be hydrated and form connected ionic channels upon humidification. In contrast, the darker zones correspond to regions with higher hydrophobicity composed of non-functionalized copolymer or functionalized copolymer whose content in sulfonic groups is low. Consequently, these regions do not participate actively in ion conduction.

The water uptake of the membranes was evaluated as a function of RH. Membranes in acidic form were vacuum-dried at 60 °C for 48 h, weighted, and placed in a climatic chamber KMF 115 (Binder GmbH) at 80 °C for 120 h and different RHs. The water uptake of the membranes was calculated in weight percent (WU%) using the following expression

(4) $$\mathrm{WU}\%=\frac{W_{\mathrm{wet}}-W_{\mathrm{dry}}}{W_{\mathrm{dry}}}\times 100$$

where $W_{\mathrm{wet}}$ and $W_{\mathrm{dry}}$ are the weights of the wet and dry membranes, respectively. Using the WU data, the volume fraction of water was determined as

(5) $$\phi_{v}=\frac{V_{w}}{V_{\mathrm{dry}}+V_{w}}=\frac{\mathrm{WU}/\rho_{w}}{1/\rho_{\mathrm{dry}}+\mathrm{WU}/\rho_{w}}$$

where $V_{w}$ ($\rho_{w}$) and $V_{\mathrm{dry}}$ ($\rho_{\mathrm{dry}}$) are the volume (density) of water and dry polymer material, respectively.

The proton conductivity was also measured at 80 °C in the range RH% = 10–100 by means of electrochemical impedance spectroscopy (EIS), using a Material Mates 7260 frequency response analyzer. The analysis was performed in a test cell composed of two gold electrodes separated by a membrane in the frequency range between 10^−1^ and 10^6^ Hz, using a voltage amplitude of 0.01 V. A Vösch 4018 climatic chamber was employed to control both temperature and relative humidity. The proton conductivity of the membranes was determined from the measured ionic resistance using the expression

(6) $$\sigma =\frac{L_{y}}{R_{m}\;A}$$

where $L_{y}$, $R_{m}$ and *A* are the thickness, ionic resistance and active area of the membrane, respectively. The experimental data obtained from the EIS measurements were analyzed using the Z-View analysis impedance software (Scribner Associates, Inc., Southern Pines, NC, USA).

## 3. Numerical Model

**Figure 3:**
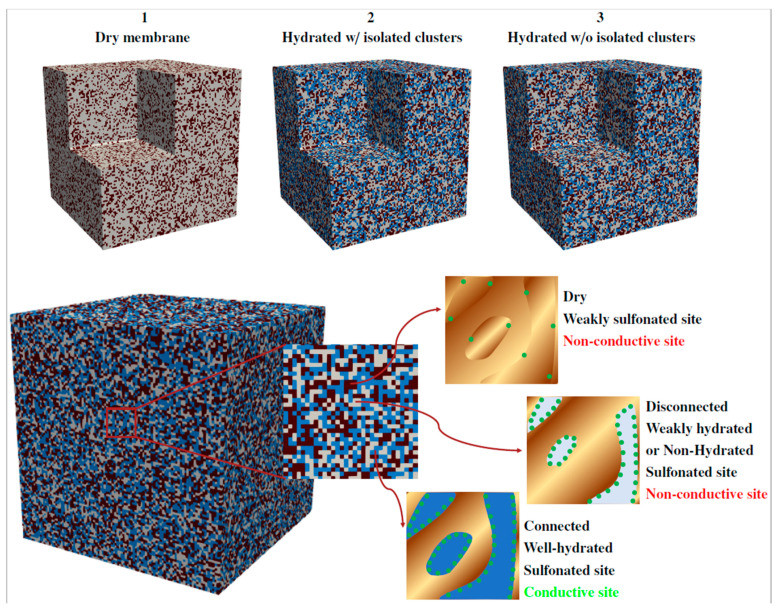
Mesoscopic representation of the copolymer membranes based on a random cubic network composed of well-hydrated sulfonated sites (type 1), weakly hydrated or non-hydrated sulfonated sites (type 2), and dry weakly sulfonated sites (type 3). Each percolation site is internally composed of a random copolymer distribution, either partially filled or not filled of water. Sulfonic groups are represented by green circles. The main steps followed for the generation of the cubic networks are illustrated at the top. Blue sites correspond to type 1, gray sites to type 2, and brown sites to type 3.

**Figure A1:**
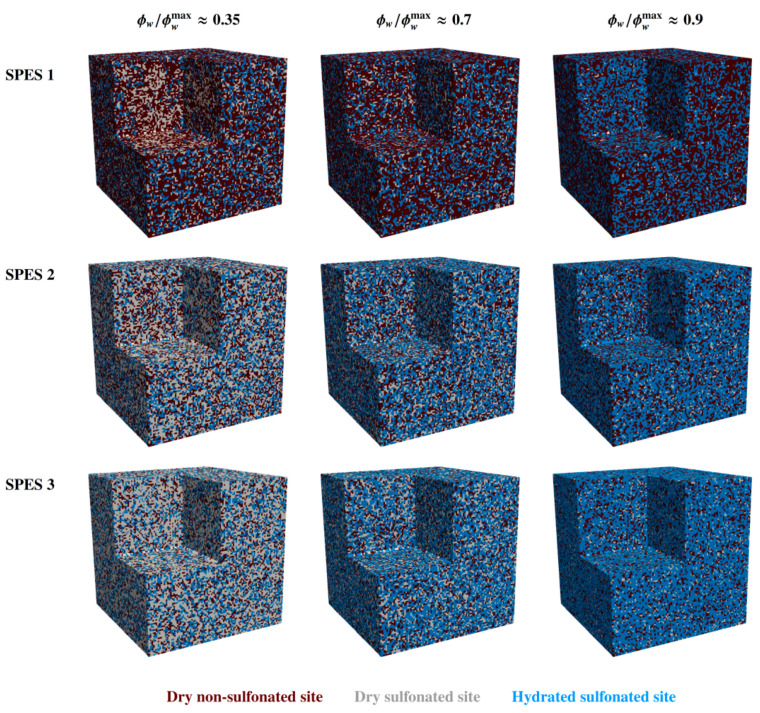
Virtually generated structures corresponding to the SPES membranes and three different relative volume fractions of hydrated sites, $\phi_{wr}/\phi_{wr}^{\max}\;\left(≃\mathrm{RH}\right)\approx 0.35,\;0.7,\;\mathrm{and}\;0.9$. Blue sites correspond to hydrated sulfonated sites, gray sites to dry sulfonated sites, and brown sites to dry non-sulfonated sites.

The ionic conductivity of the copolymer membranes was modeled using percolation theory [51]. The numerical model was implemented in the finite volume-based code ANSYS Fluent [52,53,54,55]. According to the random heterogeneous structure of the membranes observed in the FE-SEM images (see Figure 2), the structure of the membranes was divided into three types of sites in a random cubic network: (1) well-hydrated sulfonated sites, (2) weakly hydrated or non-hydrated sulfonated sites (i.e., disconnected from the water network), and (3) dry weakly sulfonated sites. Since only sites of type 1 contribute to proton conduction, sites of type 2 and 3 were not taken into account in the simulations. The mesh incorporated one computational cell per percolation site. In the mesoscopic representation of the membranes, each site is internally composed of a random copolymer distribution, either partially filled or not filled of water. Figure 3 shows an example of a virtually generated random structure, indicating the three steps followed for the creation of the cubic networks. Additional structures corresponding to the SPES membranes at different hydration levels can be found in Appendix A (Figure A1). The main steps considered in the implementation of the model are described below.

First, random numbers were generated between 0 and 1 for all sites present in the domain. Therefore, sites with a value lower than DS were labeled as sulfonated sites and those with a value higher than DS were labeled as non-sulfonated sites (type 3). The hydration level of the membranes was varied according to the relative volume fraction of hydrated or active sites, $\phi_{wr}$, defined as the ratio between the volume fraction of hydrated sites, $\phi_{w}$, and the volume fraction of sulfonated sites, DS, i.e.,

(7) $$\phi_{wr}=\;\frac{V_{h}}{V_{s}}=\frac{V_{h}/V_{t}}{V_{s}/V_{t}}=\frac{\phi_{w}}{DS}$$

where $V_{h}$ and $V_{s}$ are the volumes of hydrated and sulfonated sites, respectively, and $V_{t}$ is the total volume of the membrane. Naturally, $\phi_{wr}$ ranges between 0 and 1 and is expected to be approximately equal to RH ($\phi_{wr}≃\mathrm{RH}$). For instance, the extreme limits correspond to a fully dehydrated ($\phi_{wr}=0$) and hydrated ($\phi_{wr}=1$) membrane.

In a second step, random numbers were again generated between 0 and 1 for all the sulfonated sites identified in the previous step, so that sulfonated sites with a value lower than $\phi_{wr}$ were labeled as hydrated sulfonated sites (type 1) and the remaining sulfonated sites as non-hydrated sulfonated sites (type 2). At this point, the network contained isolated water clusters as a result of the random generation process. To mimic experimental conditions, isolated hydrated sites not connected to the edges of the domain were removed in order to form a continuous water network. Neighbor sites were identified using a six-connected voxels connectivity criterion. The isolated water clusters removed in this step were labeled as non-hydrated sulfonated sites (type 2). This operation reduced the value of $\phi_{wr}$ with respect to the one prescribed originally. Therefore, hereafter we refer to $\phi_{wr,ic}$ as the value corresponding to the network with isolated clusters, and we simply refer to $\phi_{wr}$ to the value in the final network. The maximum $\phi_{wr}$ values reached in the generated networks were 0.956, 0.999, and 0.999 for SPES 1, SPES 2 and SPES 3, respectively. Consequently, the relative humidity was considered to be approximately equal to $\mathrm{RH}≃\phi_{wr}/\phi_{wr}^{\max}$ in the networks without isolated clusters, so that both variables ranged between 0 and 1.

As commented before, in the mesoscopic model, each hydrated site is assumed to be partially filled of water. The average local volume fraction of water, $\varepsilon_{w}$, can be interpreted as the “intrinsic water-filled porosity” and, by definition, is bounded between 0 and 1; $\varepsilon_{w}$ is given by

(8) $$\phi_{v}=\;\frac{V_{w}}{V_{\mathrm{dry}}+V_{w}}=\frac{V_{s}}{V_{\mathrm{dry}}+V_{w}}\frac{V_{h}}{V_{s}}\frac{V_{w}}{V_{h}}\approx DS\phi_{wr}\varepsilon_{w}=\phi_{w}\varepsilon_{w}\Rightarrow \;\varepsilon_{w}=\frac{\phi_{v}}{\phi_{w}}$$

where $\phi_{v}$ is the experimentally determined volume fraction of water, and $V_{w}$ and $V_{\mathrm{dry}}$ are the volumes of water and polymer material, respectively.

Proton transport at the mesoscopic scale was modeled using the Nernst–Planck and charge conservation equations [56]. According to the Nernst–Planck equation, the flux of protons $H^{+}$ is given by

(9) $$N_{H^{+}}=-D_{H^{+}}\nabla C_{H^{+}}+uC_{H^{+}}-\frac{D_{H^{+}}z_{H^{+}}F}{RT}C_{H^{+}}\nabla \varphi$$

where $D_{H^{+}}$ is the (local effective) bulk diffusion coefficient of protons, $C_{H^{+}}$ is the proton concentration, $z_{H^{+}}=1$ is the charge of a proton, φ is the electrostatic (i.e., ionic) potential, and F is Faraday’s constant.

Considering that electroneutrality holds in the membranes, $C_{H^{+}}$ can be assumed equal to the fixed concentration of sulfonic groups ${\mathrm{SO}}_{3}^{-}$ (i.e., $C_{H^{+}}\approx IEC\rho_{\mathrm{wet}}$) [57]. For a constant proton concentration and negligible convection (u=0), the Nernst–Planck equation is reduced to

(10) $$N_{H^{+}}=-\frac{D_{H^{+}}F}{RT}C_{H^{+}}\nabla \varphi$$

Introducing this expression in the charge conservation equation

(11) $$\nabla \cdot j=0,\;\;j=FN_{H^{+}}$$

gives

(12) $$\nabla \cdot \left(-\frac{D_{H^{+}}F^{2}}{RT}C_{H^{+}}\nabla \varphi \right)=0\Rightarrow \nabla \cdot \left(-\sigma_{o}\nabla \varphi \right)=0$$

where $\sigma_{o}=u_{o}F^{2}C_{H^{+}}\geq 0$ is the (local effective) bulk ionic conductivity within the hydrated sulfonated (i.e., active) sites of the membrane, with $u_{o}=D_{H^{+}}/RT$ the (local effective) bulk ionic mobility of protons according to the Nernst–Einstein relation. The local ionic current density j is equal to

(13) $$j=-\sigma_{o}\nabla \varphi ,$$

with the protonic flux directed from high to low electrostatic potentials, that is, from the positive to the negative terminal of the electric circuit.

Since the bulk ionic mobility quantifies proton transfer within each hydrated site in the mesoscopic model, a reference bulk ionic conductivity $\sigma_{o}$ was assumed for SPES 3 ($\sigma_{o,3}=240{\;\mathrm{mS}\;\mathrm{cm}}^{-1}$) based on the experimental data. Then, the bulk ionic conductivity of the other two membranes was determined according to their proton concentrations, $C_{H^{+}}=IEC\rho_{\mathrm{wet}}$, where $\rho_{\mathrm{wet}}=\phi_{v}\rho_{w}+\left(1-\phi_{v}\right)\rho_{\mathrm{dry}}$ is the density of the membrane under partially saturated conditions according to the rule of mixtures. The dry density of the PSU/PPSU copolymer membranes, $\rho_{\mathrm{dry}}\approx$ $1140{\;\mathrm{kg}\;m}^{-3}$, is similar to that of water at 80 °C, $\rho_{w}=971.8{\;\mathrm{kg}\;m}^{-3}$, so that the dilution effect of water can be neglected in a first approximation. For instance, considering the maximum volume fraction of water measured for SPES 3, $\phi_{v}\approx 0.25$, the density slightly decreases to $\rho_{\mathrm{wet}}=1098{\;\mathrm{kg}\;m}^{-3}$ (3.7% lower). The estimated mean proton concentration of SPES 3 is $C_{H^{+},3}^{\mathrm{avg}}=IEC_{3}\rho_{\mathrm{wet},3}^{\mathrm{avg}}=1847\;{\mathrm{mol}\;m}^{-3}$, while the mean proton concentrations (and bulk ionic conductivities) of SPES 2 and SPES 1 are $C_{H^{+},2}^{\mathrm{avg}}=IEC_{2}\rho_{\mathrm{wet},2}^{\mathrm{avg}}=1664\;{\mathrm{mol}\;m}^{-3}$ ($\sigma_{o,2}=\sigma_{o,3}\left(C_{H^{+},2}^{\mathrm{avg}}/C_{H^{+},3}^{\mathrm{avg}}\right)=216.3{\;\mathrm{mS}\;\mathrm{cm}}^{-1}$) and $C_{H^{+},1}^{\mathrm{avg}}=IEC_{1}\rho_{\mathrm{wet},1}^{\mathrm{avg}}=1106\;{\mathrm{mol}\;m}^{-3}$ ($\sigma_{o,1}=\sigma_{o,3}\left(C_{H^{+},1}^{\mathrm{avg}}/C_{H^{+},3}^{\mathrm{avg}}\right)=143.7{\;\mathrm{mS}\;\mathrm{cm}}^{-1}$), respectively. This leads to a proton diffusion coefficient in the hydrated, conductive sites equal to $D_{H^{+}}\approx 4\times 10^{-5}{\;\mathrm{cm}}^{2}{\;s}^{-1}$, which is comparable to that reported before for fully humidified Nafion and aromatic copolymer membranes [48,58,59,60]. This value is lower than that reported for bulk liquid water at 80 °C ($D_{H^{+},w}\approx 10^{-4}\;{\mathrm{cm}}^{2}{\;s}^{-1}$) [61,62,63]. As a result, it can be inferred that both proton hopping (Grotthus mechanism) and en masse diffusion (vehicular mechanism) are important contributors to proton transfer in the vapor-equilibrated copolymer membranes [64,65,66,67].

**Figure 4:**
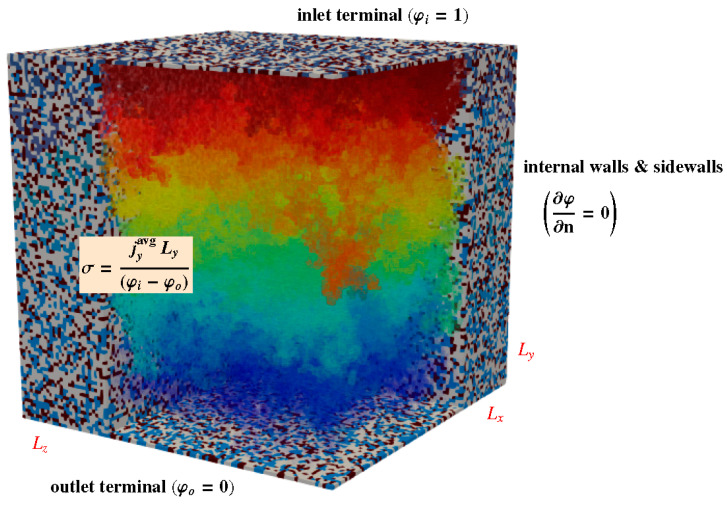
Schematic of the computational domain, showing the generated mesoscopic membrane structure and the computed ionic potential distribution, φ(x,y,z), across the hydrated, conductive network. The boundary conditions, the dimensions of the domain, and the expression used to calculate the global ionic conductivity of the network, σ, are indicated. Blue sites correspond to hydrated sulfonated sites, gray sites to weakly hydrated or non-hydrated sulfonated sites, and brown sites to dry weakly sulfonated sites.

As shown in Figure 4, the global effective (or equivalent) conductivity of the membranes, σ, was determined using volume averaging theory. The global conductivity in i-direction (for example, i=y) is given by

(14) $$j_{y}^{\mathrm{avg}}=\sigma \frac{\Delta \varphi }{L_{y}}\Rightarrow \sigma =\frac{j_{y}^{\mathrm{avg}}\;L_{y}}{\Delta \varphi };\;\Delta \varphi =\varphi_{i}-\varphi_{o}$$

where $\varphi_{i}$ and $\varphi_{o}$ are the prescribed ionic potentials at the inlet and outlet terminals, respectively, and $L_{y}$ is the length of the computational domain in y-direction. Since only hydrated sites are conductive, the volume-average ionic current density, $j_{y}^{\mathrm{avg}}$, can be calculated as

(15) $$j_{y}^{\mathrm{avg}}=\frac{1}{V_{t}}\int_{V_{t}}^{}j_{y}\;dv=DS\phi_{w}\int_{V_{h}}^{}j_{y}\;dv$$

where $V_{t}$ is the total volume of the membrane and $V_{h}$ is the volume of hydrated, conductive sites.

No flux boundary conditions were imposed at the sidewalls of the domain and the internal surfaces delimiting the hydrated, conductive network:

(16) $$\frac{\partial \varphi }{\partial n}=0$$

where n is the unit normal vector.

The size of the computational domain was set to 100 × 100 × 100 sites, since this size was found to be representative of the material. No noticeable variations in the results were observed compared to larger networks with 150 × 150 × 150 sites. It is worth mentioning that no physical length units are used in the model, so the length of each side is assumed to be $L_{x}=L_{y}=L_{z}=N_{s}\;l.u.$, where $N_{s}$ is the number of sites in each spatial direction. This consideration agrees with the FE-SEM images, where a random heterogeneous distribution of hydrophilic and hydrophobic domains was found at different length scales. In fact, the normalized global ionic conductivity, $\sigma /\sigma_{o}$, is only a function of the volume fraction of hydrated sites, $\phi_{w}$, for sufficiently large computational domains, i.e.,

(17) $$\frac{\sigma }{\sigma_{o}}=f\left(\phi_{w}\right),$$

so that the physical units of the global ionic conductivity, σ, are the same as those of the bulk ionic conductivity, $\sigma_{o}$.

## 4. Results and Discussion

**Figure 5:**
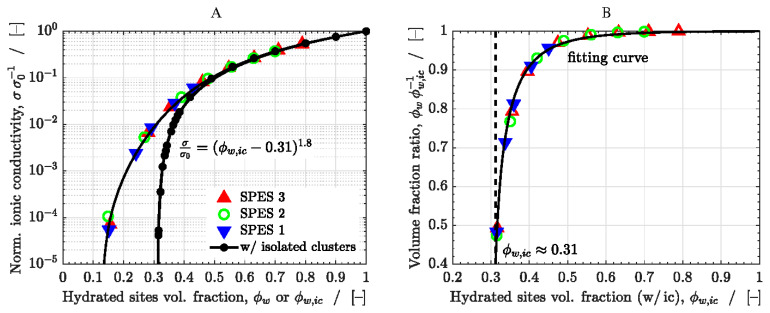
(**A**) Normalized ionic conductivity, $\sigma /\sigma_{o}$, as a function of the volume fraction of hydrated, conductive sites, $\phi_{w}=DS\phi_{wr}$, corresponding to the network without and with isolated water clusters (colored and black symbols, respectively). (**B**) Ratio between the volume fraction of hydrated, conductive sites of both networks, $\phi_{w}/\phi_{w,ic}$, as a function of $\phi_{w,ic}$. The results of the network without isolated clusters are presented for the three SPES membranes. The fitting curves to the data are included in all cases.

The computed results in dimensionless terms are shown in Figure 5. Figure 5A shows the normalized ionic conductivity, $\sigma /\sigma_{o}$, for the cubic network with and without isolated water clusters as a function of the volume fraction of hydrated, conductive sites, $\phi_{w}$, whereas Figure 5B shows the volume fraction ratio, $\phi_{w}/\phi_{w,ic}$, as a function of $\phi_{w,ic}$. The results of the network without isolated clusters are presented for the three membranes under study.

The results of the network with isolated clusters are in excellent agreement with the classical result for site percolation in a random cubic network [42,51,68], thus validating the adequate functioning of the model. The percolation threshold is $\phi_{w,ic}^{th}\approx 0.31$, while the power-law exponent is approximately equal to 1.8, leading to the percolation-type correlation

(18) $$\frac{\sigma }{\sigma_{o}}={\left(\phi_{w,ic}-0.31\right)}^{1.8}$$

This expression can be taken as a reference to map the results of the network without isolated water clusters. The ratio between the volume of hydrated sites before and after removing the isolated clusters in the network, $V_{h}/V_{h,ic}$, is a function of the original volume of hydrated sites present in the network, $V_{h,ic}$, and the total volume of the computational domain, $V_{t}$, i.e.,

(19) $$V_{h}=f\left(V_{h,ic},\;V_{t}\right)$$

Therefore, using dimensional analysis, we obtain

(20) $$\frac{V_{h}}{V_{h,ic}}=f\left(\frac{V_{h,ic}\;}{V_{t}}\right)\Rightarrow \;\frac{\phi_{w}}{\phi_{w,ic}}=f\left(\phi_{w,ic}\right)$$

As shown in Figure 5B, the relationship between $\phi_{w}$ and $\phi_{w,ic}$ can be well fitted with an exponential function of the form

(21) $$\phi_{w}=\phi_{w,ic}\frac{1-A\exp\left[{\left(\phi_{w,ic}-\phi_{w,ic}^{th}\right)}^{B}\right]}{1-A\exp\left[{\left(1-\phi_{w,ic}^{th}\right)}^{B}\right]}$$

where the dimensionless constants from the fitting are A=9 and B=0.56.

The ratio $\phi_{w}/\phi_{w,ic}$ tends to 1 when $\phi_{w,ic}≳0.5$ due to the high connectivity of the random network. However, when $\phi_{w,ic}≲0.5,\;\phi_{w}/\phi_{w,ic}$ starts to drop due to the increase of the number of isolated clusters. Eventually, when $\phi_{w,ic}\approx \phi_{w,ic}^{th}$, the water network becomes almost disconnected from the edges of the domain and $\phi_{w}/\phi_{w,ic}$ drops sharply to 0.

For a given $\phi_{w,ic}$, the normalized ionic conductivity, $\sigma /\sigma_{o}$, can be obtained from Equation (18), while the corresponding value of $\phi_{w}$ can be obtained from Equation (21). The resulting fitting curve is added to Figure 5A, showing good agreement with the computed results of the network without isolated clusters (colored symbols). As expected, when $\phi_{w,ic}≳0.5$, $\sigma /\sigma_{o}$ tends to the results of the network with isolated clusters, since both networks are virtually the same. However, when $\phi_{w,ic}≲0.5$, the results deviate from each other. The percolation threshold is reached for lower values in the network without isolated clusters due to the reduction of the volume fraction of hydrated sites compared to the network with isolated clusters.

**Figure 6:**
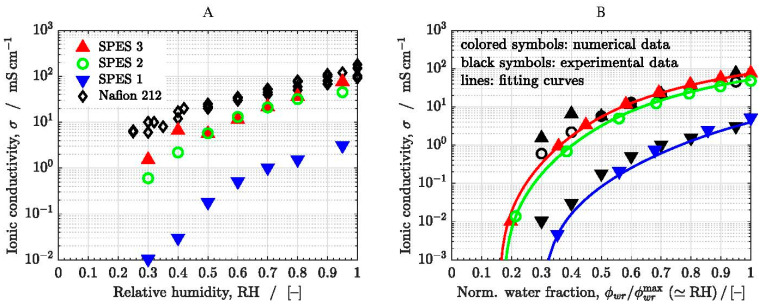
(**A**) Measured ionic conductivity as a function of relative humidity, RH, at T=80 °C of the SPES membranes. The data corresponding to Nafion 212 are also included for comparison purposes [20,69,70,71]. (**B**) Computed global ionic conductivity of the membranes (colored symbols) as a function of the ratio $\phi_{wr}/\phi_{wr}^{\max}\;\left(≃\mathrm{RH}\right)$. The experimental data (black symbols) are included for comparison purposes.

Figure 6A shows the experimental ionic conductivity measured for the three SPES membranes (see data in Table 2). The data reported in different literature sources for Nafion 212 at 80 °C are also included for comparison purposes [21,69,70,71]. Nafion 212 has a similar thickness to Nafion 112 and SPES membranes (≈50 μm), although the cell performance of Nafion 212 is somewhat better [72]. As can be seen, the ionic conductivity of SPES 3 (DS=0.79) is slightly higher than that of SPES 2 (DS=0.7), whereas the ionic conductivity of SPES 1 (DS=0.45) is significantly lower compared to the other two membranes. It drops around two orders of magnitude in the full RH range examined (RH = 0.3–0.95). The conductivity of SPES 3 is similar to that of Nafion 212 at high relative humidity (RH≳0.7−0.8). For instance, Ureña et al. [24] reported similar ionic conductivities in single cell measurements performed with membrane electrode assemblies, including SPES 3 and Nafion 112, at 80 °C and 100% RH ($\sigma_{\mathrm{MEA},3}\approx 29.8\;{\mathrm{mS}\;\mathrm{cm}}^{-1}$ vs. $\sigma_{\mathrm{MEA},112}\approx 34.3\;{\mathrm{mS}\;\mathrm{cm}}^{-1}$). Note that the ionic conductivities were reduced by one third compared to those measured ex-situ due to the additional ionic resistance of the catalyst layers and interfaces [73,74]. However, the ionic conductivity of the copolymer membranes is lower than that of Nafion 212 at low relative humidity (RH≲0.7). This fact can be ascribed to differences in the morphology and the DS of the membranes (DS=1 for Nafion), as well as to the different dependence of the ionic concentration on water uptake ($\rho_{\mathrm{dry},\mathrm{SPES}}\approx 1.14\;{\mathrm{kg}\;m}^{-3}\;\mathrm{vs}.\;\rho_{\mathrm{dry},212}\approx 2.05\;{\mathrm{kg}\;m}^{-3}$). This aspect is further analyzed below with the numerical model.

The nonlinear dependence of the ionic conductivity on DS can be explained based on percolation theory using the dimensionless results presented previously, see Figure 6. Introducing $\phi_{w,ic}=DS\phi_{wr,ic}$ in Equation (18), we have that

(22) $$\sigma =\sigma_{o}{\left(DS\phi_{wr,ic}\;-0.31\right)}^{1.8}$$

where $\phi_{wr,ic}$ can be related to $\phi_{wr}$ using Equation (21).

Considering $\phi_{wr,ic}\approx \phi_{wr}\approx 1$, the ratio between the ionic conductivity of SPES 1 ($DS_{1}=0.45$, $IEC_{1}=0.97{\;\mathrm{meq}\;g}^{-1}$) and SPES 3 ($DS_{3}=0.79$, $IEC_{3}=1.62{\;\mathrm{meq}\;g}^{-1}$) is

(23) $$\frac{\sigma_{1}}{\sigma_{3}}\approx \frac{\sigma_{o,1}}{\sigma_{o,3}}{\left(\frac{DS_{1}-0.31}{DS_{3}-0.31}\right)}^{1.8}=\frac{IEC_{1}}{IEC_{3}}{\left(\frac{DS_{1}-0.31}{DS_{3}-0.31}\right)}^{1.8}=0.6\times 0.1\sim 10^{-2}$$

while the percolation threshold of both membranes differs by a factor of about two:

(24) $$\frac{\phi_{wr,1}^{th}}{\phi_{wr,3}^{th}}=\frac{\phi_{wr,ic,1}^{th}}{\phi_{wr,ic,3}^{th}}=\frac{DS_{3}}{DS_{1}}=1.75$$

The differences between SPES 2 and SPES 3 are smaller due to their similar DS ($DS_{2}=0.7$ vs. $DS_{3}=0.79$) and IEC ($IEC_{2}=1.46\;{\mathrm{meq}\;g}^{-1}$ vs. $IEC_{3}=1.62\;{\mathrm{meq}\;g}^{-1}$).

**Figure 7:**
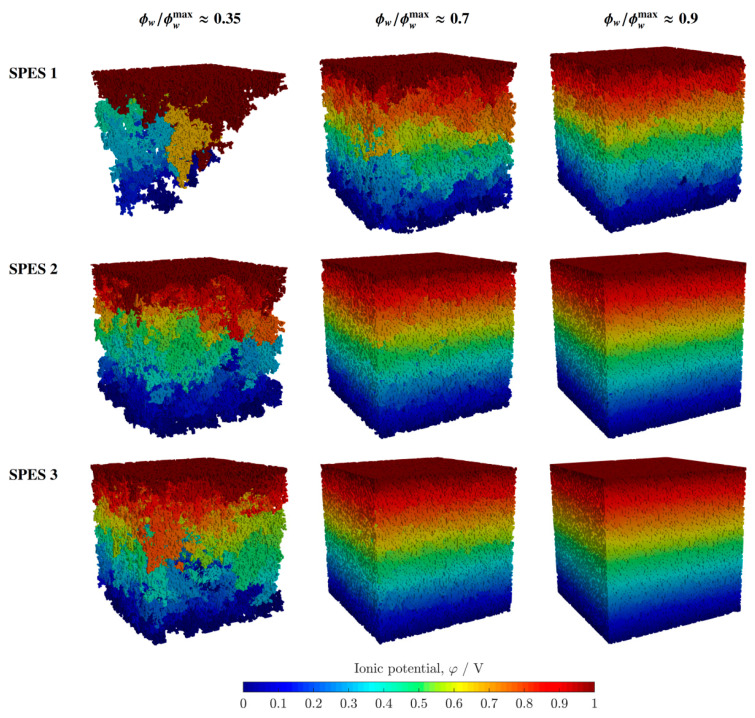
Computed ionic potential distributions corresponding to the SPES membranes and three different relative volume fractions of hydrated sites, $\phi_{wr}/\phi_{wr}^{\max}\;\left(≃\mathrm{RH}\right)\approx 0.35,\;0.7,\;\mathrm{and}\;0.9$.

The ionic potential distributions of the three SPES membranes corresponding to various relative volume fractions of hydrated sites ($\phi_{wr}/\phi_{wr}^{\max}\;\left(≃\mathrm{RH}\right)\approx 0.35,\;0.7,\;\mathrm{and}\;0.9$) are shown in Figure 7. As can be seen, the conductive networks of SPES 2 and SPES 3 are rather similar in the full RH range, in agreement with the results presented before in Figure 6. In contrast, the hydrated, conductive networks of SPES 1 are significantly more disconnected. For instance, when $\phi_{wr}/\phi_{wr}^{\max}\approx 0.35,$ the ionic network of SPES 1 approaches the percolation threshold, so that only a few pathways connect both extremes of the membrane. As a result, proton transport is strongly hindered, and the ionic conductivity decreases dramatically.

The effect of the interconnectivity of the percolation networks on proton conduction can be further analyzed using the following expression for the normalized ionic conductivity [7,8]:

(25) $$\frac{\sigma }{\sigma_{o}}=\frac{\phi_{w}}{\tau }\Rightarrow \tau =\frac{\phi_{w}}{\sigma /\sigma_{o}}$$

where $\phi_{w}$ is the volume fraction of hydrated sites and τ is the (conduction) tortuosity factor; $\phi_{w}$ accounts for the linear decrease of the conductivity that would result for straight conductive domains connected in parallel (i.e., with a tortuosity factor equal to 1, $L_{y}^{\mathrm{eff}}=L_{y}$), whereas τ accounts for the reduction of the conductivity caused by the existence of no straight and dead-end ionic pathways, i.e., $\tau ={\left(L_{y}^{\mathrm{eff}}/L_{y}\right)}^{2}$, where $L_{y}^{\mathrm{eff}}$ is the effective length of transport pathways and $L_{y}$ is the straight length across the membrane.

**Figure 8:**
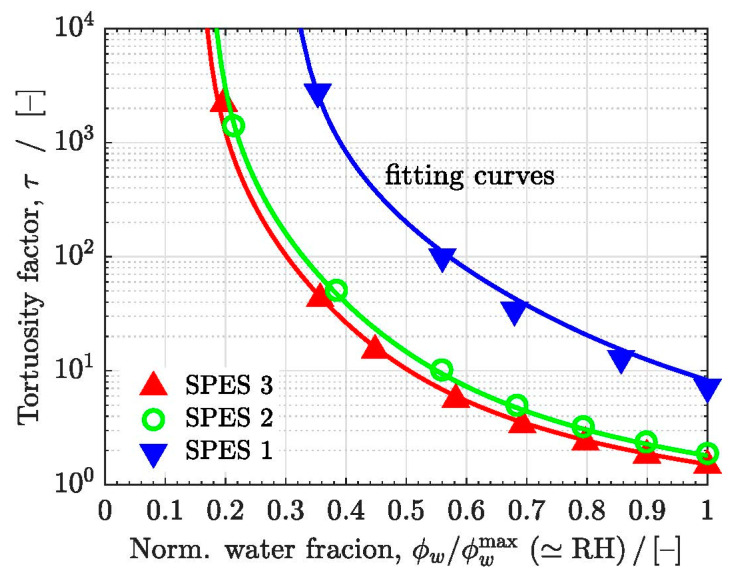
Tortuosity factor τ as a function of the ratio $\phi_{w}/\phi_{wr}^{\max}$ (≃RH ) of the SPES membranes. The fitting curves to the data are included.

The computed tortuosity factors of the three SPES membranes are shown in Figure 8 as a function of $\phi_{wr}/\phi_{wr}^{\max}\;\left(≃\mathrm{RH}\right)$, together with the fitting curves obtained using Equations (22) and (25). For the three SPES membranes, the tortuosity factor grows as the hydration of the membranes decreases, increasing sharply when RH is close to the percolation threshold. The tortuosity factors of SPES 1 are around one order of magnitude higher than those of SPES 2 and SPES 3 ($\tau_{1}∼10\;\tau_{2},\tau_{3}$). Therefore, since $\phi_{w}$ is similar for the three membranes ($\phi_{w,1}∼\phi_{w,2}∼\phi_{w,3}$), the remaining difference in the conductivity of the membranes arises from the reduction of the intrinsic bulk conductivity ($\sigma_{o,1}∼10^{-1}\sigma_{o,2},\sigma_{o,3}\;$); see Equation (23). Both $\sigma /\sigma_{o}$ (i.e., tortuosity) and $\sigma_{o}$ (i.e., ionic concentration and mobility) play an important role on the global conductivity, σ. Here, it is worth noting the multiscale nature of proton conduction in PEMs, since the tortuosity and size of ionic channels within each hydrated site affects in turn the value of $\sigma_{o}$ through the (local effective) proton diffusivity, $D_{H^{+}}$. As shown in previous works, the membrane processing conditions significantly affect the multiscale morphology of block copolymer membranes [75,76,77,78,79,80]. For instance, Assumma et al. [75] reported a ten-fold increase in proton conductivity after thermal annealing of SPAS multiblock copolymer membranes. This was ascribed to a beneficial impact of annealing on (i) the co-continuous nanophase separated morphology, (ii) the local arrangement of neighboring side chains and/or improved connectivity of ionic channels, and (iii) favorable organization of conductive domains at higher scales.

**Figure 9:**
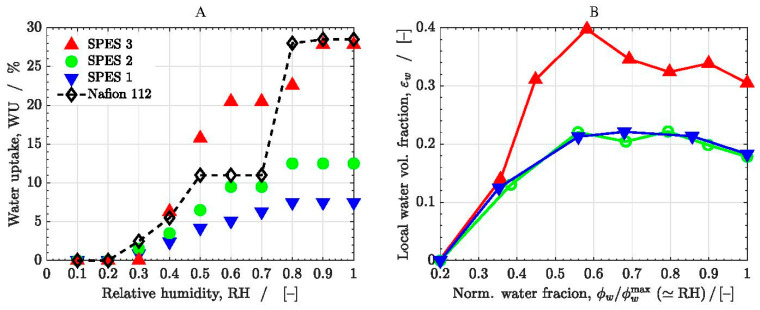
(**A**) Measured water uptake, WU%, as a function of relative humidity, RH, at T=80 °C of the SPES membranes. The results for Nafion 112 are also included. (**B**) Predicted local water volume fraction, $\varepsilon_{w}$, as a function of the ratio $\phi_{w}/\phi_{wr}^{\max}$ (≃RH ).

Figure 9 shows the measured WU% of the three SPES membranes as a function of RH, together with the data measured for Nafion 112. The corresponding data are summarized in Table 2. As expected, the WU% of the membranes increases with RH and DS. The difference in the WU% of SPES 1 and SPES 2 is small, even though the DS of both membranes is significantly different. In contrast, the WU% of SPES 3 is notably higher compared to that of SPES 2, despite their similar DS. The WU% reached with SPES 3 is similar to that of Nafion 112. This nonlinear behavior is explained by the gradual sulfonation of the PPSU block as the average DS is increased (see Table 1). In the case of SPES 3, the DS of both units is nearly the same ($DS_{\mathrm{PPSU}}=0.82\approx DS_{\mathrm{PSU}}=0.76$), so there is actually no microphase separation in the random co-continuous copolymer structure [24]. A similar effect was reported in previous works for copolymer membranes with an increasing length of the hydrophilic unit due to better percolation of hydrophilic domains with increasing sulfonated block length (see, e.g., [22,75,76]).

**Figure A2:**
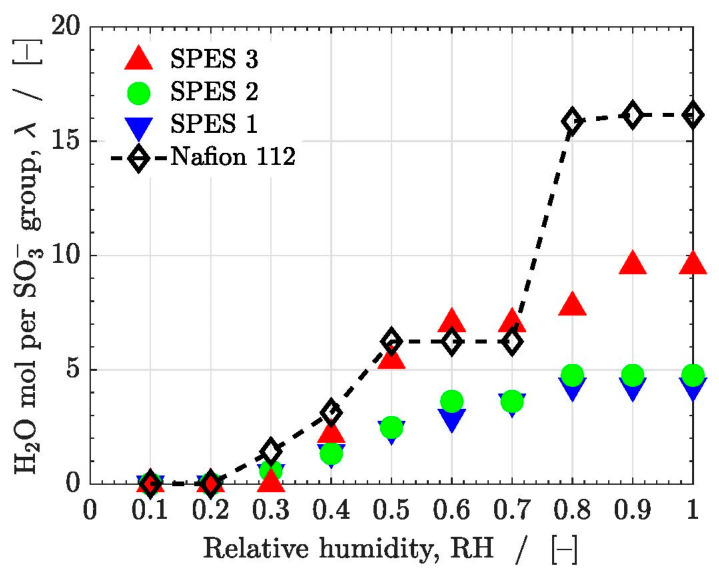
Measured water content (water molecules per sulfonic acid group (molecule)), λ, as a function of relative humidity RH of the SPES membranes. The results for Nafion 112 are also included ($IEC=0.98\;{\mathrm{meq}\;g}^{-1}$ [49]).

These observations agree with the mesoscopic representation of the membranes adopted in the model, where there is a strong increase of the local volume fraction of water, $\varepsilon_{w}$, between SPES 3 and SPES 2. For a given RH value (RH≳0.5), this is explained by the increase of the volume fraction of water, $\phi_{v}$, between both membranes, while the volume fraction of hydrated sites, $\phi_{w}$, remains approximately equal (i.e., $\varepsilon_{w}=\phi_{v}/\phi_{w}$ increases for SPES 3). However, in the case of SPES 1 and SPES 2, $\varepsilon_{w}$ is rather similar because $\phi_{v}$ and $\phi_{w}$ grow rather proportionally (i.e., $\varepsilon_{w}=\phi_{v}/\phi_{w}\approx \mathrm{cte}.$). As shown in Appendix A (Figure A2), the water content (molecules) per sulfonic acid group, $\lambda =\mathrm{WU}/\left(IECM_{w}\right)$, shows a similar trend to WU% and $\varepsilon_{w}$. Thus, a higher hydration number prevails for SPES 3 compared to SPES 1 and SPES 2. Nevertheless, it should be pointed out that the higher hydration number of SPES 3 does not significantly affect the global ionic conductivity. The reason behind this behavior is due to the similar density of the copolymer membranes and water, such that the dilution caused by water on the ionic concentration is negligible. The slight increase of the global conductivity of SPES 3 is mainly ascribed to the increase of $\sigma_{o}$ owing to (i) the increment of IEC, and/or (ii) the contribution from the increase of the size and better interconnectivity of ionic channels with water within each hydrated, conductive site (an effect not taken into account explicitly in our simplified model) [75,76,77].

**Figure 10:**
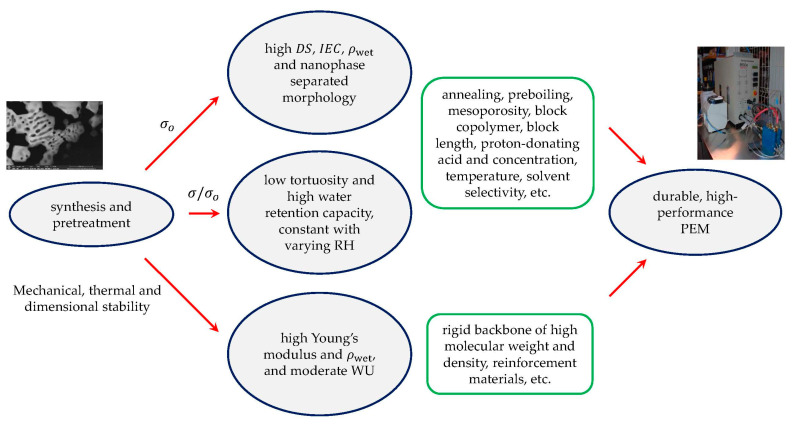
Flow chart showing key variablesand synthesis parameters for the design of durable, high-performance proton-exchange membranes (PEMs) extracted from the numerical model and experiments.

The modeling results and experimental data can be used to extract general guidelines for the design of high-conductivity copolymer membranes in a wider RH range (see Figure 10). As commented before, to achieve high proton conductivity, it is necessary to increase both $\sigma /\sigma_{o}$ and $\sigma_{o}$. The former ($\sigma /\sigma_{o}$) can be increased by synthesizing membranes with high DS (high interconnectivity of sulfonated sites) and low tortuosity (ideally, τ=1). The latter ($\sigma_{o}$) can be increased by synthesizing membranes with high ionic concentration (i.e., high wet density and IEC, such as doubly functionalized polymers [81]) and high (effective) proton diffusivity (i.e., good co-continuous nanoscale phase separation with low-tortuosity ionic channels).

In order to achieve good performance at low RH, it is also necessary to have low tortuosity values (ideally, τ=1  at any length scale and RH), such that $\sigma /\sigma_{o}$ only decreases linearly with the volume fraction of water [75]. This linear decrease can be in turn mitigated by designing PEMs with a high water retention capacity (i.e., a constant volume fraction of water, ideally $\phi_{v}\approx \mathrm{cte}.,\;\phi_{w}\approx 1$, with varying RH). For instance, previous works showed a superior conductivity of Nafion membranes by incorporating mesopores into the (porous) polymer structure [82,83,84]. The benefit of this practice could be related to an increase of the interfacial surface of the membrane exposed to the humidified environment (i.e., an increase of the number of “points of water sorption”) and to a better interconnectivity of ionic channels introduced by water-filled mesopores. Moreover, an increase of the ionic concentration with decreasing RH may serve to counterbalance the reduction of the ionic conductivity caused by a lower water content. For this, it would be necessary to synthesize membranes with densities notably higher than that of water (e.g., Nafion has twice the density of water).

In summary, membranes with high IEC, DS and $\rho_{\mathrm{wet}}$, as well as with a low-tortuosity multiscale morphological structure, are good candidates for high proton conduction. These requirements must be accompanied by a good control of water uptake (water volume uptake notably lower than volume of the dry membrane) and high mechanical and thermal stability, using, for example, rigid polymer backbones of high molecular weight and/or reinforcement materials for extended durability [22,24,85,86,87,88,89].

## 5. Conclusions

In this work, proton conduction in membranes based on multiblock copolymer of sulfonated PSU/PPSU poly(ether sulfone)s was examined experimentally and numerically. Three membranes with different average degrees of sulfonation (DS) and ion-exchange capacities (IEC) were analyzed: SPES 1 ($DS_{1}=0.45$, $IEC_{1}=0.97$ ${\mathrm{meq}\;g}^{-1}$), SPES 2 ($DS_{2}=0.70$, $IEC_{2}=1.46{\;\mathrm{meq}\;g}^{-1}$), and SPES 3 ($DS_{3}=0.79$, $IEC_{3}=1.62{\;\mathrm{meq}\;g}^{-1}$). The corresponding DS of each copolymer block were $DS_{\mathrm{PPSU}}=0.18,\;0.61,\;0.8$ and $DS_{\mathrm{PSU}}=0.75,\;0.81,\;0.76$ for SPES 1, SPES 2, and SPES 3, respectively.

The morphology of the membranes was visualized by field emission scanning electron microscopy (FE-SEM), while the water uptake and ionic conductivity were measured as a function of relative humidity (RH) at 80 °C by weight difference and electrochemical impedance spectroscopy, respectively. The FE-SEM images showed a random heterogeneous distribution of hydrophilic and hydrophobic domains, with high and low contents of sulfonic groups, respectively. The structure of the membranes was reproduced using a mesoscopic model formulated on a random cubic network. Proton transport through the percolation network was modeled using simplified Nernst–Planck and charge conservation equations.

The nonlinear increase of the conductivity with RH and DS was successfully explained using percolation theory. The ionic conductivity of SPES 2 and SPES 3 ($\sigma ∼1-100{\;\mathrm{mS}\;\mathrm{cm}}^{-1}$) increased around two orders of magnitude compared to SPES 1 ($\sigma ∼10^{-2}-1{\;\mathrm{mS}\;\mathrm{cm}}^{-1}$) in the range RH=0.3−1 due to the higher DS and IEC of the former membranes, whereas the percolation threshold of SPES 2 and SPES 3 (${\mathrm{RH}}^{th}\approx 0.18$) decreased by a factor of two compared to SPES 1 (${\mathrm{RH}}^{th}\approx 0.32$). Physically, the decrease of the ionic conductivity of the SPES membranes with decreasing RH and DS is affected by two factors: (i) the decrease of the volume of ionic channels available for transport, and (ii) the higher tortuosity of transport pathways. In addition, the water uptake of the three membranes varied nonlinearly. The water uptakes of SPES 1 and SPES 2 were nearly the same, despite their different DS. However, the water uptake of SPES 3 increased strongly, even though the DS was close to that of SPES 2. In agreement with previous works, this behavior was explained by the similar DS of both copolymer blocks ($DS_{\mathrm{PPSU}}\approx DS_{\mathrm{PSU}}$) and the lack of microphase separation in the random co-continuous copolymer structure of SPES 3. Similar conclusions were obtained from the mesoscopic model, where a strong rise of the local water volume fraction was predicted for SPES 3 compared to SPES 1 and SPES 2 due to the increase of the volume fraction of water-to-volume fraction of hydrated sites ratio.

Based on the numerical results and experimental observations, general guidelines for the design of durable, high-performance copolymer membranes were extracted. Proton-exchange membranes with high IEC, DS, wet density and nanophase-separated morphology, as well as a low-tortuosity multiscale structure and high water retention capacity, are good candidates for high proton conduction in a wider RH range. Meanwhile, mechanical, thermal, and dimensional stability must be preserved using rigid polymer backbones of high molecular weight and/or reinforcement materials.

Future work should consider the improvement of the model to include thermal effects and a more comprehensive description of proton transfer within ionic channels, including relevant variables, such as heterogeneous pore size distribution, and spacing and arrangement of sulfonic groups. Furthermore, the results presented here should be considered to assist the design of next-generation copolymer membranes with improved properties. Additional research lines include the application of the model to analyze the ionic conductivity, gas permeability and permselectivity of copolymer membranes in other electrochemical systems, such as membranes for vanadium redox flow batteries and thin ionomer films in catalyst layers, among others.

## Figures and Tables

**Table 1:** Ion-exchange capacity (IEC) and average degree of sulfonation (DS) of the SPES membranes, together with the DS of each block copolymer. The IECs of Nafion 112 and Nafion 117 are also included.

Membrane	$IEC/\mathrm{meq}\;g^{-1}$	$DS_{\mathrm{PPSU}}$	$DS_{\mathrm{PSU}}$	DS
SPES 1 [24]	0.97	0.18	0.75	0.45
SPES 2 [24]	1.46	0.61	0.81	0.70
SPES 3 [24]	1.62	0.82	0.76	0.79
Nafion 112 [48,49]	0.90, 0.98	-	-	-
Nafion 117 [50]	0.93	-	-	-

**Table 2:** Water uptake, WU%, and ionic conductivity, σ, of the SPES membranes and Nafion 112 at 80 °C and different RH%. As indicated in brackets, the last measurement of the ionic conductivity was performed at RH% = 95.

RH%	SPES 1 WU% $\sigma_{m}$ $\mathrm{mS}\;cm^{-1}$		SPES 2 WU% $\sigma_{m}$ $\mathrm{mS}\;cm^{-1}$		SPES 3 WU% $\sigma_{m}$ $\mathrm{mS}\;cm^{-1}$		Nafion 112 WU%
10	0	-	0	-	0	-	0
20	0	-	0	-	0	-	0
30	0.9	0.01	1.5	0.6	0	1.53	2.5
40	2.4	0.03	3.5	2.19	6.3	6.6	5.5
50	4.2	0.18	6.5	5.73	15.75	5.64	11.0
60	5.1	0.51	9.5	13.02	20.48	11.4	11.0
70	6.3	1.02	9.5	20.97	20.48	21.75	11.0
80	7.5	1.53	12.5	31.86	22.57	36.63	28
90 (95)	7.5	(3.09)	12.5	(45.24)	27.82	(77.2)	28.5
100	7.5	-	12.5	-	27.82	-	28.5

## Nomenclature

A	area$/m^{2}$: dimensionless constant in Equation (21)/-
B	dimensionless constant in Equation (21)/-
C	species concentration$/mol\;m^{-3}$
IEC	ion-exchange capacity$/mol\;kg^{-1}$
D	species diffusivity/ $m^{2}\;s^{-1}$
DS	degree of sulfonation (i.e., percentage of sulfonated copolymer blocks)/-
F	Faraday’s constant/ $C\;mol^{-1}$
j	ionic current density$/A\;m^{-2}$
L	length/m
M	molecular mass$/kg\;mol^{-1}$
m	number of structural units of the PPSU block/-
$m_{dry}$	mass of dry membrane/kg
$m_{p}$	mass of sulfonated copolymer per mol of dry sulfonated copolymer$/kg\;mol^{-1}$
N	number of moles per mol of dry sulfonated copolymer/-
$N_{s}$	number of sites in each spatial direction of the computational domain/-
N	molar flux/ $mol\;m^{-2}\;s^{-1}$
n	number of structural units of the PSU block/-
n	unit normal vector/-
R	universal gas constant/ $J\;mol^{-1}\;K^{-1}$
$R_{m}$	membrane ionic resistance/Ω
T	temperature/K
u	ionic mobility$/mol\;s\;kg^{-1}$
u	velocity$/m\;s^{-1}$
V	volume$/m^{3}$
W	weight/N
WU	water uptake/-
$z_{i}$	charge of species i
*Greek letters*	
$\varepsilon_{w}$	average local volume fraction of water/-
λ	hydration number (number of water molecules per sulfonic group)/-
ρ	density$/kg\;m^{-3}$
σ	global proton conductivity$/S\;m^{-1}$
$\sigma_{o}$	bulk proton conductivity$/S\;m^{-1}$
τ	tortuosity factor/-
$\phi_{v}$	volume fraction of water/-
$\phi_{w}$	volume fraction of hydrated or active sites/-
$\phi_{wr}$	relative volume fraction of hydrated or active sites/-
φ	ionic potential/V
*Subscripts*	
b	polymer backbone
dry	dry membrane
h	hydrated site
i	inlet
ic	isolated clusters
o	outlet or bulk value
s	sulfonated site
$SO_{3}^{-}$	sulfonic group
t	total
w	water
wet	humidified condition
x	x-direction in the material plane (first in-plane direction)
y	thickness direction
z	z-direction in the material plane (second in-plane direction)
*Superscripts*	
avg	average
th	threshold
max	maximum
*Abbreviations*	
FC	fuel cell
PEMFC	proton-exchange membrane fuel cell
PEM	proton-exchange membrane
PFSA	perfluorinated sulfonic acid
RH	relative humidity
(S)PAE	(sulfonated) poly(arylene ether)
PAS	poly(arylene sulfone)
SPESK	sulfonated poly(arylene ether sulfone ketone)
(S)PSU	(sulfonated) polysulfone
(S)PPSU	(sulfonated) polyphenylsulfone
FE-SEM	field emission scanning electron microscopy
SPES	sulfonated poly(ether sulfone)
SPPO	sulfonated poly(phenylene oxide)

## Appendix A

The virtually generated structures of the SPES membranes for three different relative volume fractions of hydrated sites, $\phi_{wr}/\phi_{wr}^{\max}\;\left(≃\mathrm{RH}\right)\approx 0.35,\;0.7,\;\mathrm{and}\;0.9$, are shown in Figure A1. As can be seen, the number of hydrated, conductive sites (blue sites) increases with $\phi_{wr}/\phi_{wr}^{\max}\;\left(≃\mathrm{RH}\right)$ for the three membranes. The number of dry non-sulfonated sites (brown sites) is significantly higher for SPES 1 ($DS_{1}=0.45$), while it is rather similar for SPES 2 and SPES 3 ($DS_{2}=0.7$ and $DS_{3}=0.79$).

Figure A2 shows the measured water content (water molecules per sulfonic acid group), $\lambda =\mathrm{WU}/\left(IECM_{w}\right)$, as a function of relative humidity, RH, of the SPES membranes, including also the results for Nafion 112. The hydration numbers of SPES 1 and SPES 2 are rather similar, while it grows significantly for SPES 3 ($\lambda_{3}\approx 10\;\mathrm{vs}.\;\lambda_{1}\approx \lambda_{2}\approx 5$ at fully humidified condition). The hydration number of Nafion 112 is much higher than that of SPES 3 at fully humidified condition due to its lower IEC (0.98 vs. 1.62) and similar WU% (28%) [21,49].
